# Supplementary material for: miR-1199-5p and Zeb1 function in a double-negative feedback loop potentially coordinating EMT and tumour metastasis
Source: Nat Commun. 2017 Oct 27;8:1168. doi: 10.1038/s41467-017-01197-w (PMC5660124; doi:10.1038/s41467-017-01197-w)
Supplement: Supplementary file 1 — Supplementary information [file 41467_2017_1197_MOESM1_ESM.pdf]

Supplemental Information

Supplemental Figures

Supplementary Figure 1

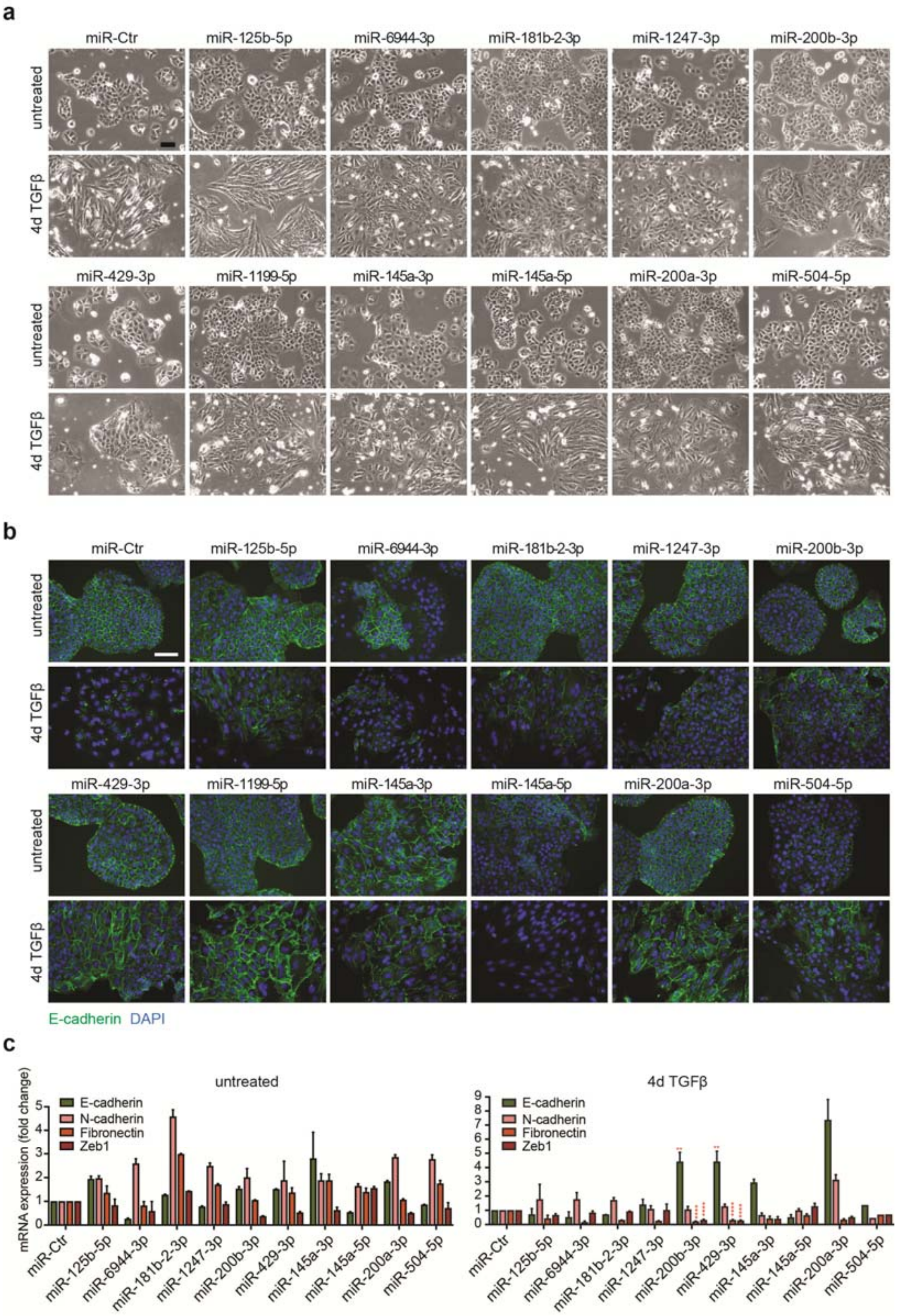

Supplementary Fig. 1 Phenotypic screen for miRNAs affecting a TGFβ-induced EMT.

(a) Bright field images of NMuMG/E9 cells transfected with miRNA mimics as indicated. NMuMG/E9 cells were cultured in the absence (*top*) or presence of TGFβ (4 days; *bottom*). Scale bar: 100μm.

(b) Immunofluorescence microscopy images of NMuMG/E9 cells cultured and treated as described in (a) and stained for the epithelial marker E-cadherin (*green*). DAPI was used to visualise nuclei. Scale bar: 100μm.

(c) Epithelial (E-cadherin) and mesenchymal (N-cadherin, Fibronectin, Zeb1) gene expression was analysed by quantitative RT-PCR in NMuMG/E9 cells cultured and treated as described in (a). Data are presented as mean fold changes +/- s.e.m.; n=2 (miR-504-5p: n=1; miR-200b/429-3p 4d TGFβ: n=4); significance determined by an unpaired, two-sided t-test; **P < 0.01, ****P < 0.0001.

Supplementary Figure 2

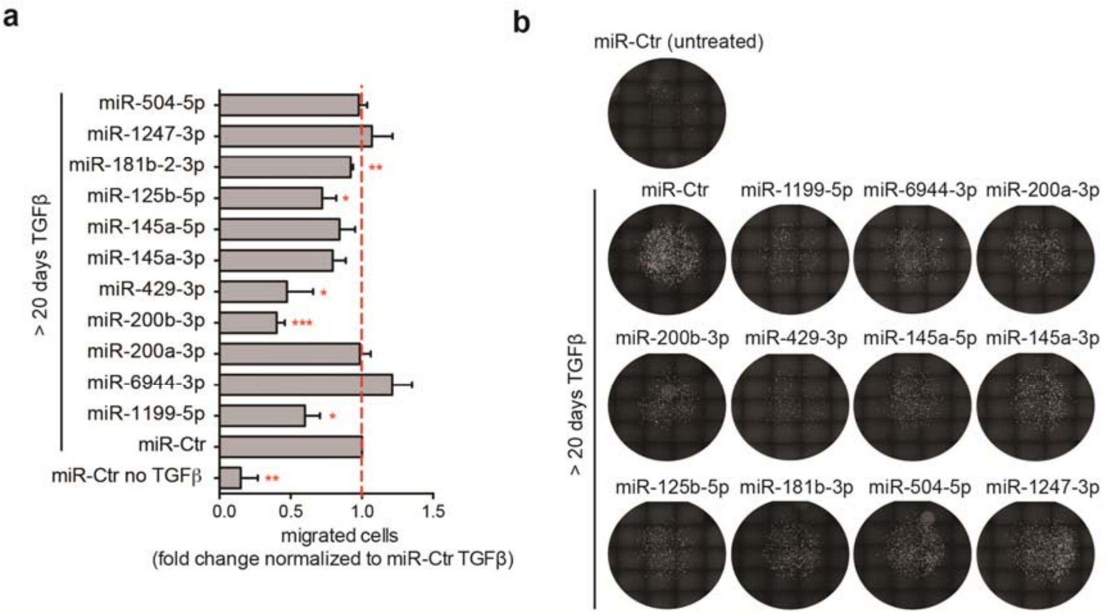

Supplementary Fig. 2 Identification of miRNAs regulating mesenchymal tumour cell migration.

(a) Quantification of migrated epithelial (no TGFβ) and mesenchymal (> 20 days TGFβ) Py2T cells transfected with the miRNA mimics indicated in a 96-well plate format. The graph summarizes the number of migrated cells per insert normalized to the total cell number (input plate). Data were normalized to miR-Ctr transfected cells (+TGFβ; red line) and are presented as mean fold

changes +/- s.e.m.; n=3, significance determined by an unpaired, two-sided t-test *P < 0.05, **P < 0.01, ***P < 0.001.

(b) Representative immunofluorescence images of migrated epithelial (untreated) and mesenchymal (> 20 days TGFβ) Py2T cells from the experiment described in (a). Nuclei were visualized with DAPI staining and the bottom of the trans-well migration inserts were imaged by fluorescence microscopy.

Supplementary Figure 3

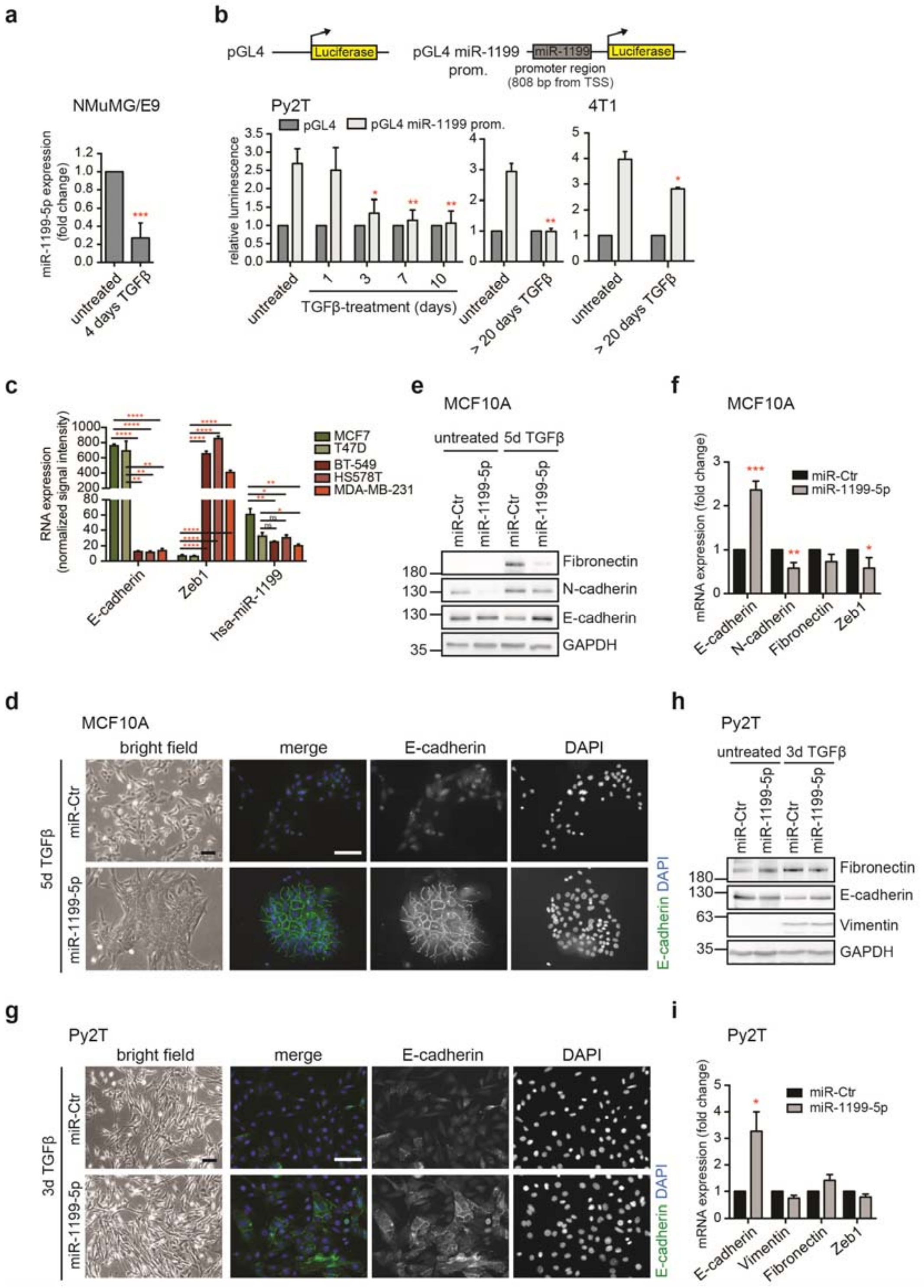

Supplementary Fig. 3 Ectopic expression of miR-1199-5p affects EMT and cell migration.

(a) NMuMG/E9 cells were cultured in the absence (untreated) and presence of TGFβ (4 days TGFβ). miRNA-1199-5p expression levels were analysed by

RT-PCR (mean fold changes +/- s.e.m.; n=4; significance determined by an unpaired, two-sided t-test ***P < 0.001).

(b) *Top*: Schematic representation of the miR-1199 promoter luciferase reporter constructs. *Bottom:* Py2T (*left*) and 4T1 (*right*) cells were treated with TGFβ for the time points indicated. Cells were transfected with a *Renilla* luciferase reporter along with either a miR-1199-promoter *Firefly* luciferase-reporter (pGL4 miR-1199 promoter) or a control reporter (pGL4) construct. Relative luminescence (*Firefly*/*Renilla*) was calculated and normalized to the control reporter (mean fold changes +/- s.e.m.; n=3; significance determined by an unpaired, two-sided t-test *P < 0.05, **P < 0.01).

(c) Hsa-miR-1199 expression analysis in human breast cancer cell lines. RNA expression (normalized array signal intensity) profiles of E-cadherin, Zeb1 and miR-1199 in epithelial (green) and mesenchymal (red) breast cancer cell lines.

(d-f) Ectopic expression of human miR-1199-5p blocks TGFβ-induced EMT in MCF10A cells. (d) Representative bright field and immunofluorescence images of MCF10A cells transfected with the miRNA mimics indicated. Cells were cultured in the presence of TGFβ for 5 days. Localization of E-cadherin was analysed by immunofluorescence microscopy. DAPI was used to stain nuclei. Scale bar: 100µm. (e) Immunoblot analysis for the EMT markers indicated on the cells described in (d). GAPDH was used as loading control. (f) RT-PCR analysis for the EMT markers indicated was examined in the cells described in (d). Data are presented as mean fold changes +/- s.e.m.; n=3; significance determined by an unpaired, two-sided t-test *P < 0.05, **P < 0.01, ***P < 0.001.

(g-i) Ectopic expression of miR-1199-5p delays TGFβ-induced EMT in Py2T cells. Cell morphology and E-cadherin immunofluorescence staining (g) and EMT marker expression analyses by immunoblotting (h) and RT-PCR (i) were performed as described for MCF10A cells in (d-f).

Supplementary Figure 4

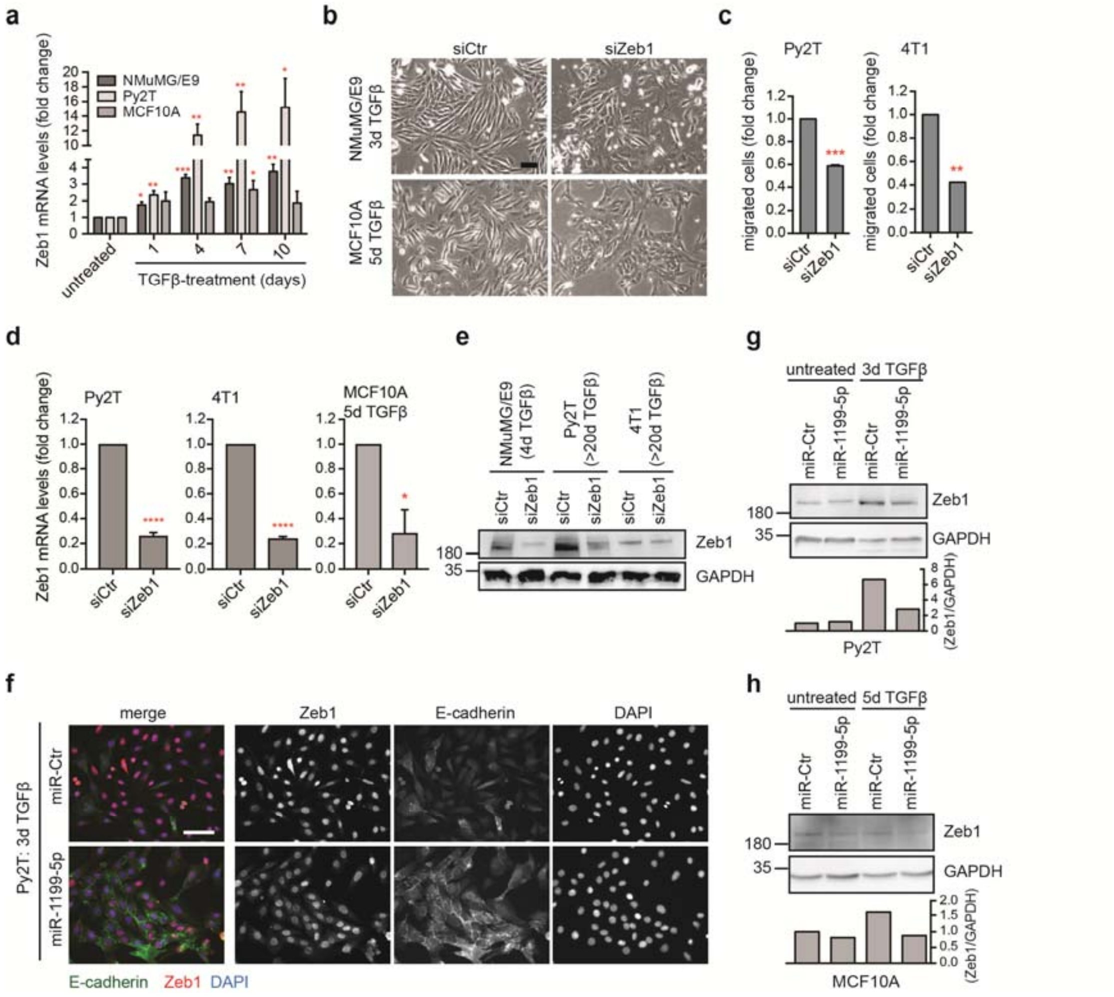

Supplementary Fig. 4 Regulation and function of Zeb1 during an EMT.

(a) Zeb1 transcript levels increase upon a TGFβ-induced EMT in different cellular systems. mRNA levels of Zeb1 were determined by RT-PCR analysis in NMuMG/E9, Py2T and MCF10A cells treated with TGFβ for the time points indicated. Data present mean fold changes +/- s.e.m.; n=3; for MCF10A 4d TGFβ: n=2; significance determined by an unpaired, two-sided t-test *P < 0.05, **P < 0.01, ***P < 0.001**.**

(b) Loss of Zeb1 blocks TGFβ-induced EMT. Bright field images illustrate morphological differences of NMuMG/E9 (*top*) and MCF10A (*bottom*) cells transfected with a siRNA against Zeb1 or a negative control. Cells were cultured in the presence of TGFβ for 4 (NMuMG/E9) or 5 days (MCF10A). Scale bar: 100µm.

(c) Loss of Zeb1 reduces cancer cell migration. Mesenchymal (> 20 days TGFβ) Py2T (*left*) and 4T1 (*right*) cells were transfected with the siRNAs indicated and plated in a FCS gradient of a Boyden chamber trans-well migration insert. After 18 hours, trans-migrated cells per insert were quantified. Results present mean fold changes +/- s.e.m.; n=3; significance determined by an unpaired, two-sided t-test **P < 0.01, ***P < 0.001.

(d,e) Analysis of Zeb1 knockdown efficiencies. Mesenchymal Py2T and 4T1 cells as well as MCF10A cells and NMuMG/E9 cells were transfected and cultured as described in (b,c). Quantitative RT-PCR (mean fold changes; n=3; significance determined by an unpaired, two-sided t-test *P < 0.05, ****P < 0.0001.) (d) and immunoblotting analyses (e) were used to analyse siRNA-mediated knockdown of Zeb1.

(f-h) Forced expression of miR-1199-5p reduces Zeb1 protein levels. Py2T and MCF10A cells were transfected with a miR-Ctr or mmu-miR-1199-5p (Py2T) or hsa-miR-1199-5p (MCF10A) mimic and cultured in the absence (untreated) or presence of TGFβ (Py2T: 3 days; MCF10A: 5 days). Immunofluorescence analysis in Py2T cells (f) and immunoblotting analysis in Py2T (g) and MCF10A (h) cells were used to assess Zeb1 protein levels. (g,h) Top: immunoblotting analysis. Bottom: quantification of Zeb1 protein levels (fold change) from immunoblotting and subsequent normalization to GAPDH protein levels. Scale bar: 100µm.

Supplementary Figure 5

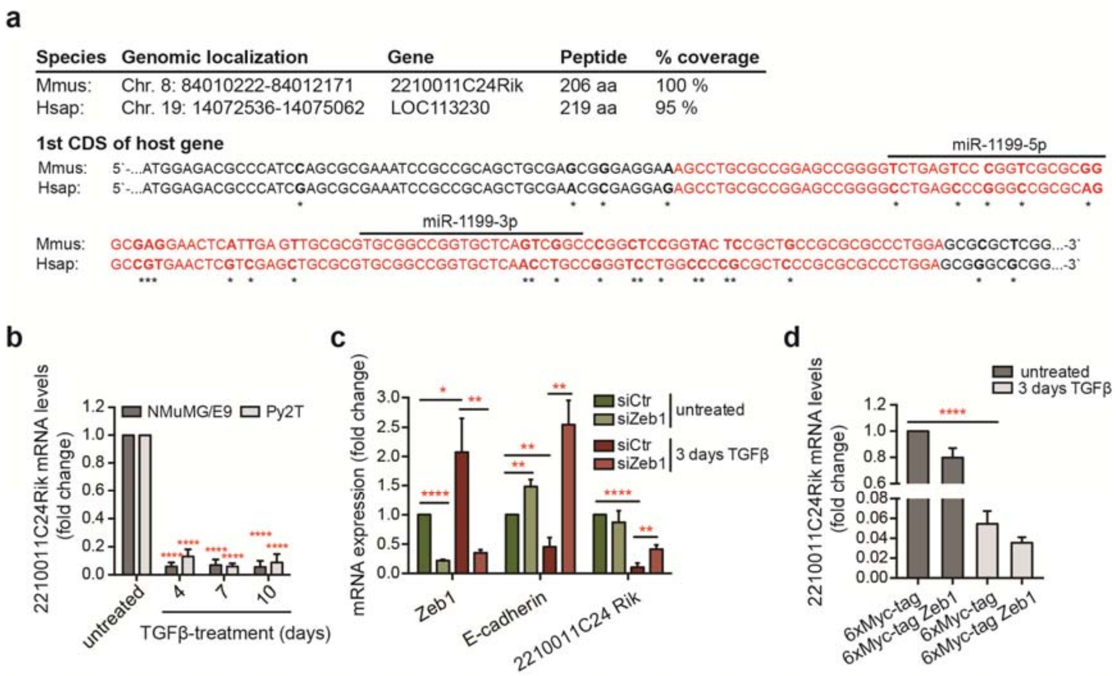

Supplementary Fig. 5 The miR-1199 host gene 2210011C24Rik: Co-regulation and conserved genomic localization.

(a) *Top*: Localization, peptide length (aa) and cDNA similarities (coverage in %) of miR-1199 host genes in the human (Hsap) and murine (Mmus) genome. *Bottom*: Sequence alignment of murine and human miR-1199 gene sequences (*red*) within the first CDS of its host gene. Differences in nucleotides within both sequences are marked with * and miR-1199-5p/-3p sequences within the miR-1199 gene are highlighted.

(b) 2210011C24Rik transcript levels decrease during a TGFβ-induced EMT. mRNA levels of 2210011C24Rik were quantified by RT-PCR analysis in NMuMG/E9 and Py2T cells treated with TGFβ for the time points indicated. Data are presented as mean fold changes +/- s.e.m.; n = 4; significance determined by an unpaired, two-sided t-test ****P < 0.0001.

(c) Loss of Zeb1 blocks TGFβ-induced EMT and induces a delay in 2210011C24Rik mRNA downregulation. NMuMG/E9 cells were transfected with a siRNA against Zeb1 or a negative control and cultured in the absence (untreated; *green*) or presence of TGFβ (3 days; *red*). Transcript levels of Zeb1, E-cadherin and 2210011C24Rik were determined by RT-PCR analysis. Data are presented as mean fold changes +/- s.e.m.; n=3; significance

determined by an unpaired, two-sided t-test *P < 0.05, **P < 0.01, ****P < 0.0001.

(d) Forced expression of Zeb1 moderately diminishes 2210011C24Rik expression. NMuMG/E9 cells were transiently transfected with a 6xMyc-tag Zeb1 or a 6xMyc-tag control construct and cultured in the absence (untreated) or presence of TGFβ (3 days). Transcript levels of 2210011C24Rik were quantified by RT-PCR analysis (mean fold changes +/- s.e.m.; n=3; significance determined by an unpaired, two-sided t-test ****P < 0.0001).

Supplementary Figure 6

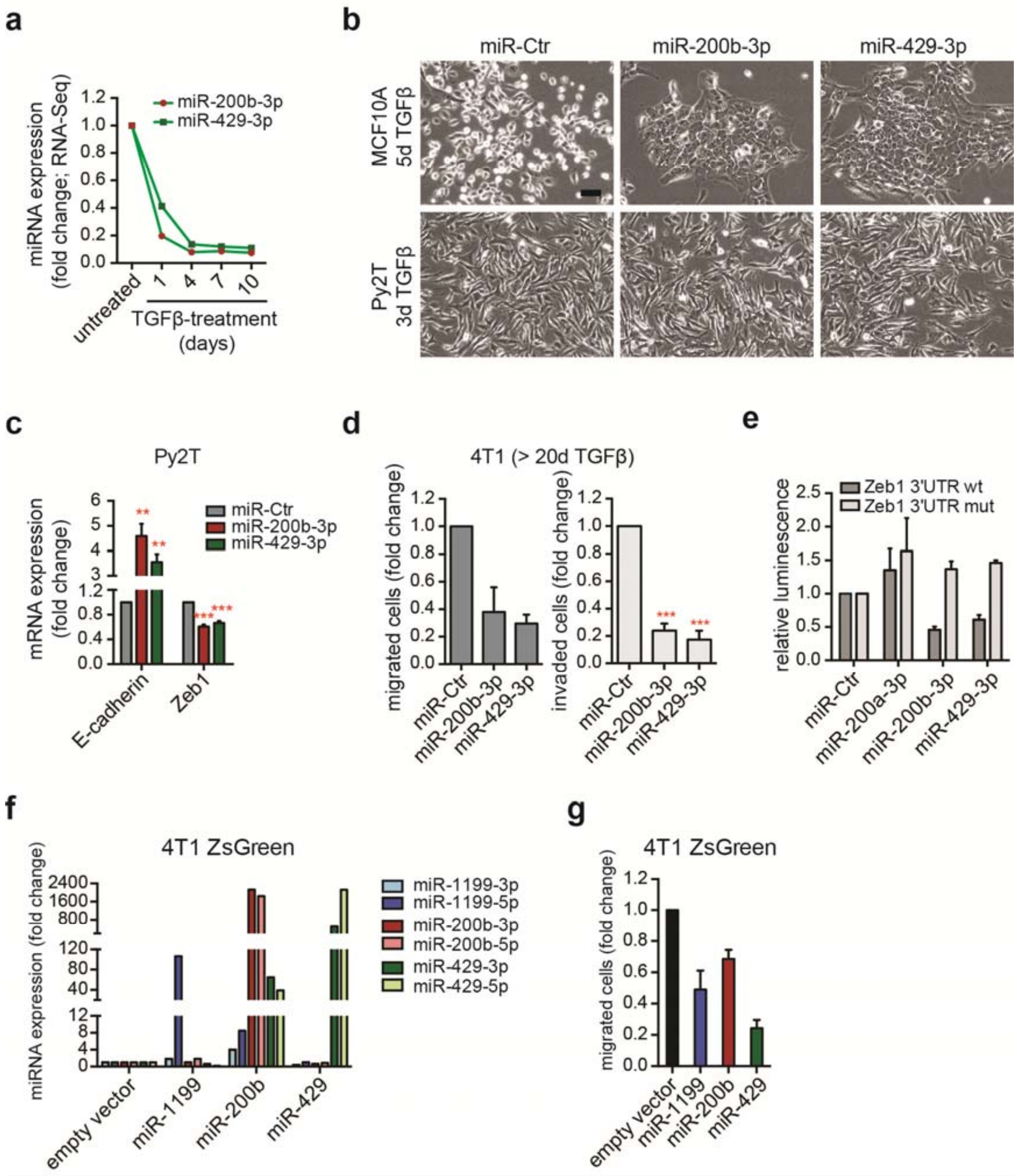

Supplementary Fig. 6 miR-200b-3p and miR-429-3p regulation and function during an EMT.

(a) MiR-200b-3p and miR-429-3p transcript levels decrease during an EMT. Expression profiles (mean fold changes) of miR-200b-3p (*red dots*) and miR-429-3p (*green squares*) during a TGFβ-induced EMT of NMuMG/E9 cells measured by RNA-sequencing analysis.

(b) Representative bright field images of MCF10A and Py2T cells transfected with the miRNA mimics indicated. Cells were cultured in the presence of TGFβ for 5 or 3 days, respectively. Scale bar: 100μm.

(c) RT-PCR analysis of EMT marker mRNA expression Py2T cells. Cells were transfected and cultured as described in (b). Data are presented as mean fold changes +/- s.e.m. (n=3; significance determined by an unpaired, two-sided t-test **P < 0.01, ***P < 0.001).

(d) Forced expression of miR-200b-3p and miR-429-3p reduces cancer cell migration and invasion. Mesenchymal 4T1 cells (> 20 days TGFβ) were transfected with the miRNA mimics indicated and plated in a FCS gradient of a Boyden chamber trans-well migration (*left*) or invasion (*right*) insert. The numbers of trans-migrated/invaded cells were quantified. Graphs represent the mean fold changes +/- s.e.m. compared to miR-Ctr transfected cells (migration: n=2; invasion: n=3; significance determined by an unpaired, two-sided t-test ***P < 0.001).

(e) Post-transcriptional regulation of Zeb1 by miR-200b-3p and miR-429-3p. NMuMG/E9 cells were transfected with the miRNA mimics indicated, a *Renilla* luciferase reporter construct and with either a wild type or a mutant Zeb1-3`UTR *Firefly* luciferase-reporter construct. Relative luminescence (*Firefly*/*Renilla*) was calculated and normalized to miR-Ctr transfected cells (mean fold changes +/- s.e.m.; n=2).

(f) Stable co-expression of ZsGreen and miR-1199, miR-200b or miR-429 or ZsGreen alone (empty vector) in 4T1 cells. Quantitative RT-PCR was used to analyse miR-1199-5p/-3p, miR-200b-3p/-5p and miR-429-3p/5p expression. Data are presented as fold changes compared to empty vector-transduced cells (n=1).

(g) Stable expression of miR-1199, miR-200b or miR-429 in 4T1 cells reduces trans-well cell migration. 4T1 cells described in (f) were treated with TGFβ for 3 days and plated within a FCS gradient on Boyden chamber migration inserts. The numbers of transmigrated cells were quantified. Depicted are mean fold changes +/- s.e.m.; n=2.

Supplementary Figure 7

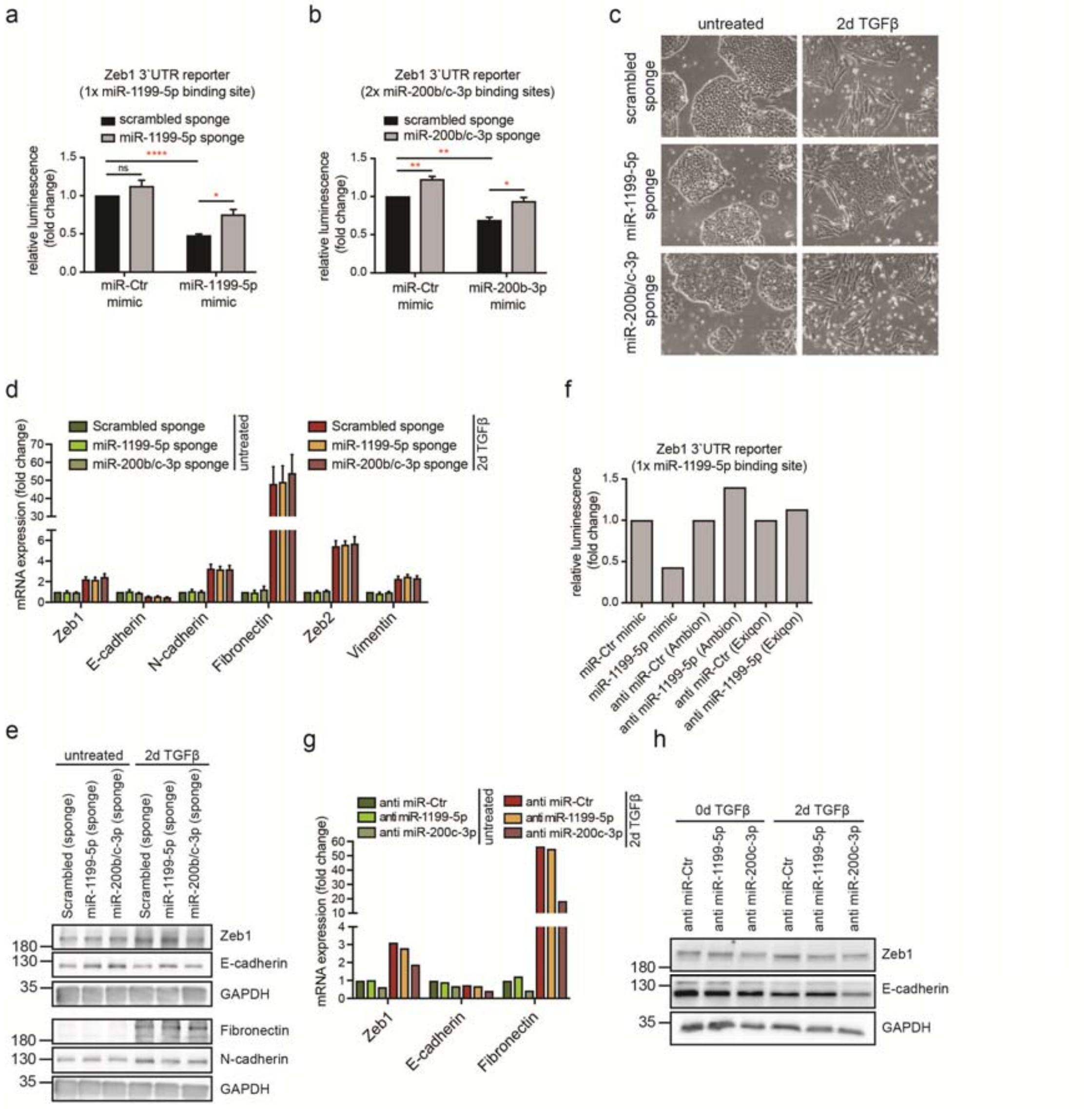

Supplementary Fig. 7 Inhibition of miR-1199-5p and miR-200b/c-3p during EMT.

(a-b) miR1199-5p and miR-200b/c-3p sponge constructs are functional. NMuMG/E9 cells were transiently transfected with a miR-1199-5p, a miR-200b-3p or a miR-Ctr mimic along with the miRNA sponge constructs indicated and with a Zeb1 3`UTR *Firefly* luciferase reporter construct exhibiting one miR-1199-5p seed sequence (a) or two miR-200b/429-3p seed sequences (b). Cells were co-transfected with a Renilla luciferase reporter and fold changes +/- s.e.m. of relative luminescence (Firefly/Renilla

luminescence) were calculated (n=3; significance determined by an unpaired, two-sided t-test *P < 0.05, **P < 0.01, ***P < 0.001).

(c-e) Loss of miR-1199-5p or miR-200b/c-3p is not sufficient to induce an EMT. NMuMG/E9 cells were transiently transfected with the miRNA sponge constructs indicated. (c) Bright field images of NMuMG/E9 cells cultured in the absence and presence of TGFβ. Quantitative RT-PCR (d; data are presented as mean fold changes +/- s.e.m.; n=3) and immunoblotting (e) analysis was performed for the expression of different EMT markers.

(f-h) NMuMG/E9 cells were transiently transfected with a miR-Ctr, miR-1199-5p or a miR-200c-3p inhibitor from two different companies (Ambion and Exiqon). (f) A Zeb1 3`UTR *Firefly* luciferase reporter with one miR-1199-5p binding site was used as described in (a) (n=1). Quantitative RT-PCR (g; data are presented as mean fold changes +/- s.e.m.; n=3) and immunoblotting (h) analyses was performed for the expression of different EMT markers.

Supplementary Figure 8

Source data, western blots

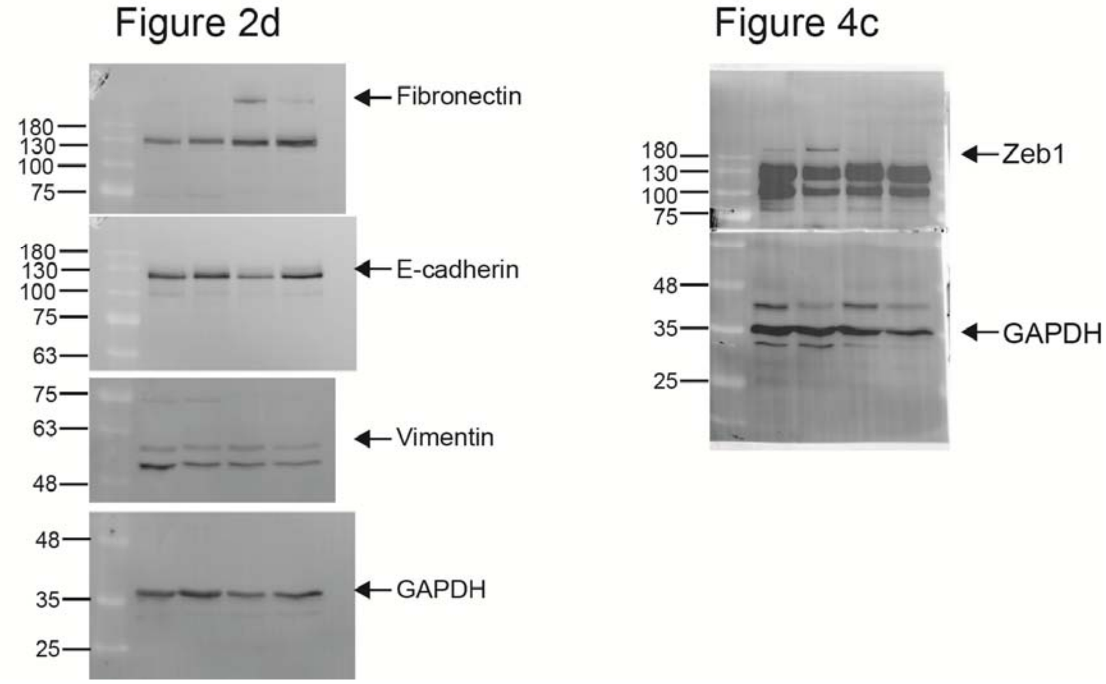

Supplementary Fig. 8 Uncropped scans from the immunblotting results presented in the main figures.

Supplementary Table 1: Predicted direct target genes of miR-1199-5p during an EMT.

Summarized are the 66 genes which are: a) predicted to be direct targets of miR-1199-5p by miRWalk2.0 (predicted targets in at least seven out of 13 prediction databases with a predicted seed sequence in the 3’UTR, 5’UTR or CDS) and b) display reduced transcript levels upon forced expression of miR-1199-5p in NMuMG/E9 cells at 4 days of TGFβ-treatment (RNA-sequencing analysis; log2FC (-1), FDR < 0.05).

	**miR-1199-5p**	
**Gene**	**log2FC**	**FDR**
Pitpnm3	-3.865	9.24E-29
Col5a1	-3.805	3.53E-31
Asic1	-3.584	2.33E-09
Chrna1	-3.532	8.61E-10
Padi3	-3.048	6.16E-19
Megf10	-2.978	1.71E-10
Pik3cd	-2.714	7.26E-17
Havcr2	-2.688	2.48E-07
Gprc5b	-2.511	1.34E-06
Gpc6	-2.424	2.49E-12
Pdgfrb	-2.397	4.49E-12
Cdk18	-2.337	1.52E-38
Spn	-2.245	4.97E-27
Sepn1	-2.234	7.08E-43
Cmah	-2.174	6.19E-05
Zeb1	-2.065	1.60E-21
Zfp9	-2.054	1.71E-14
Sox12	-2.034	2.76E-09
Il12rb1	-2.029	1.09E-07
Fbxo32	-1.810	2.15E-10
8430408G22Rik	-1.803	1.32E-02
Atp2b4	-1.767	1.51E-17
Gm14137	-1.712	2.61E-08
Slc7a11	-1.642	3.35E-04
Pkp1	-1.619	1.66E-03
Oasl2	-1.602	4.18E-07
Gpr157	-1.552	2.78E-06
Map3k1	-1.534	3.00E-08
Cds1	-1.529	1.90E-06
Pla2g16	-1.502	7.76E-07
Xdh	-1.487	1.69E-06
Plac1	-1.484	2.89E-05
Cdon	-1.471	1.50E-13

Slc35f1	-1.470	5.85E-06
St8sia1	-1.443	7.58E-05
Cdk5r1	-1.437	2.19E-06
Elovl6	-1.418	5.99E-09
Klhl14	-1.394	2.15E-02
Slc25a36	-1.383	7.32E-09
Mex3a	-1.382	6.78E-15
Ncs1	-1.364	1.48E-15
Fosl2	-1.345	1.91E-13
Sorcs2	-1.336	1.92E-03
Cd28	-1.331	3.03E-03
Nav1	-1.310	1.98E-05
Dnm1	-1.273	2.38E-03
Sall2	-1.273	1.22E-06
Foxn1	-1.254	7.20E-04
Ctdspl2	-1.235	1.26E-07
Skil	-1.213	7.34E-05
Etv4	-1.213	4.06E-07
Fhod3	-1.209	5.00E-12
Csrnp2	-1.199	3.43E-10
Tnfaip2	-1.181	6.66E-04
Pitpnc1	-1.179	4.53E-11
Cep170	-1.139	5.11E-05
Spsb4	-1.116	2.77E-06
Pdgfra	-1.088	3.44E-03
Cyp7a1	-1.082	9.11E-04
Coro2b	-1.074	5.36E-03
Akr1c14	-1.064	4.34E-02
Klhl30	-1.051	1.86E-04
Amotl1	-1.041	1.72E-05
Zmiz1	-1.033	3.43E-08
Itpripl2	-1.026	9.91E-08
Gm12185	-1.013	7.48E-03

Supplementary Table 2: Common direct target genes of miR-200b-3p and miR-429-3p during an EMT.

The table summarizes 54 genes which are a) common, predicted direct targets of miR-200b-3p and miR-429-3p by Walk2.0 (predicted targets in at least seven out of 13 prediction databases with a predicted seed sequence in the 3'UTR, 5'UTR or CDS) and b) display reduced transcript levels upon forced expression of miR-200b-3p or miR-429-3p in NMuMG/E9 cells at 4 days of TGFβ-treatment (RNA-sequencing analysis; log2FC (-1), FDR < 0.05).

	**miR-200b-3p**		**miR-429-3p**	
**Gene**	**log2FC**	**FDR**	**log2FC**	**FDR**
Tubb3	-4.031	5.51E-19	-3.961	3.71E-18
Lrp1	-3.205	3.34E-39	-3.094	7.73E-37
Msn	-3.139	4.42E-33	-2.804	3.11E-27
Loxl2	-3.113	2.55E-19	-2.863	1.49E-16
Tln2	-3.100	3.15E-39	-2.736	1.29E-31
Zeb2	-3.090	2.34E-21	-2.882	7.05E-19
Has2	-3.064	1.25E-04	-3.056	1.79E-04
Foxf2	-2.834	5.05E-47	-2.758	1.20E-44
Cd1d1	-2.817	2.38E-13	-2.145	8.32E-08
Tmprss11f	-2.771	8.03E-08	-2.726	2.30E-07
Rdh10	-2.768	1.86E-18	-2.742	3.98E-18
Zcchc24	-2.716	2.27E-09	-2.924	7.04E-11
Meox1	-2.505	4.95E-09	-2.613	1.38E-09
Pkd1	-2.363	9.63E-26	-2.312	1.08E-24
Fn1	-2.313	1.50E-19	-2.226	3.24E-18
Pcdhb16	-2.123	8.69E-14	-2.001	2.73E-12
Cyp1b1	-2.091	2.25E-30	-2.027	1.34E-28
Pthlh	-2.059	3.33E-07	-2.016	1.17E-06
Rasl12	-1.970	1.10E-06	-1.887	4.51E-06
Chsy1	-1.915	5.97E-13	-1.765	3.87E-11
Fnbp1	-1.873	6.72E-23	-1.761	2.44E-20
Sema3f	-1.839	2.55E-19	-1.600	6.49E-15
Wipf1	-1.834	7.58E-20	-1.483	2.15E-13
Cbx4	-1.829	4.45E-17	-1.610	1.82E-13
Cish	-1.810	1.09E-22	-1.944	1.34E-25
Prr9	-1.788	4.69E-07	-1.609	2.69E-05
Trps1	-1.660	1.01E-07	-1.167	3.06E-04
Efnb2	-1.644	1.94E-19	-1.672	5.65E-20
Msrb3	-1.621	1.42E-12	-1.502	7.00E-11
Nuak1	-1.613	1.78E-12	-1.471	1.61E-10
Prkab2	-1.573	6.11E-10	-1.347	1.62E-07
Gata2	-1.488	9.30E-06	-1.459	2.84E-05
Cast	-1.462	4.69E-10	-1.159	1.33E-06

Nin	-1.430	9.97E-07	-1.292	1.35E-05
Rbfox2	-1.372	2.76E-16	-1.183	2.73E-12
Extl3	-1.336	1.45E-14	-1.076	9.75E-10
Aff1	-1.333	6.60E-10	-1.044	2.31E-06
Ckap4	-1.299	1.35E-10	-1.309	1.13E-10
Fscn1	-1.299	3.13E-04	-1.187	1.49E-03
Lrrk2	-1.253	4.20E-06	-1.285	2.71E-06
Wnt1	-1.211	6.37E-04	-1.120	9.43E-03
Vash2	-1.200	4.05E-07	-1.045	1.51E-05
Blcap	-1.177	4.70E-10	-1.162	9.01E-10
Snai1	-1.143	4.94E-04	-1.269	1.07E-04
Foxn3	-1.098	7.01E-07	-1.061	2.13E-06
Nyap1	-1.083	2.09E-03	-1.013	4.88E-03
Ubxn8	-1.057	2.62E-07	-1.061	3.03E-07
Trio	-1.028	4.03E-06	-1.055	2.43E-06
Col5a1	-3.933	9.99E-33	-3.651	9.14E-29
Zeb1	-2.958	8.32E-41	-2.873	1.27E-38
Zfp9	-1.460	5.32E-08	-1.414	1.81E-07
Sox12	-1.148	1.22E-03	-1.092	2.62E-03
Cdon	-1.757	6.24E-19	-1.385	3.64E-12
Ncs1	-1.542	8.90E-20	-1.535	1.63E-19

Supplementary Table 3: All pre-miR miRNA precursors used in this study for the forced expression of miRNAs and functional screening.

**miRNA**	**Catalog number (Ambion)**
mmu-miR-1199-5p	PM13577
hsa-miR-1199-5p	PM26554
hsa/mmu-miR-200b-3p	PM10492
mmu-miR-429-3p	PM10759
hsa-miR-429-3p	PM10221
mmu-miR-145a-3p	PM12730
mmu-miR-2137	PM15897
mmu-miR-486-5p	PM10546
mmu-miR-3107-5p	PM10546
mmu-miR-181a-5p	PM10421
mmu-miR-6944-3p	PM27934
mmu-miR-145a-5p	PM11480
mmu-miR-125b-5p	PM10148
mmu-miR-206-3p	PM10409
mmu-miR-139-5p	PM12466
mmu-miR-181b-2-3p	PM20146
mmu-miR-101b-5p	PM20094
mmu-miR-181a-2-3p	PM12578
mmu-miR-125b-2-3p	PM12421
mmu-miR-143-3p	PM10883
mmu-miR-1247-3p	PM19654
mmu-miR-7a-5p	PM10047
mmu-miR-218-5p	PM10328
mmu-miR-200a-3p	PM10991
mmu-miR-210-5p	PM20577
mmu-miR-504-5p	PM12429
mmu-miR-30c-5p	PM11060
mmu-miR-1968-5p	PM14972
mmu-miR-1199-3p	PM19640
mmu-miR-200b-5p	PM12857
mmu-miR-200a-5p	PM10250
mmu-miR-802-3p	PM20311
mmu-miR-1247-5p	PM13197
mmu-miR-802-5p	PM11932

Supplementary Table 4: List of oligonucleotides used for quantitative RT-PCR

**Name**	**fwd. primer (5`- 3`)**	**rev. primer (5`- 3`)**
	Murine genes	
mRpl19	ctcgttgccggaaaaaca	tcatccaggtcaccttctca
mCdh1	cgaccctgcctctgaatcc	tacacgctgggaaacatgagc
mFibronectin	cccagacttatggtggcaatt	aatttccgcctcgagtctga
mVimentin	ccaaccttttcttccctgaa	ttgagtgggtgtcaaccaga
mZeb1	gccagcagtcatgatgaaaa	tatcacaatacgggcaggtg
mZeb2	ggaggaaaaacgtggtgaactat	gcaatgtgaagcttgtcctctt
mSox4	cctcgctctcctcgtcct	tcgtcttcgaactcgtcgt
m2210011C24Rik	agatgcaacgggacatcg	gcgcttgagttcgtccag
	Human genes	
hRpl19	gatgccggaaaaacaccttg	tggctgtacccttccgctt
hCdh1	agaacgcattgccacatacact	tctgatcggttaccgtgatcaa
hFibronectin	gaactatgatgccgaccagaa	ggttgtgcagatttcctcgt
hCdh2	tagtcaccgtggtcaaaccaat	gtgctgaattcccttggctaat
hZeb1	gccaacagaccagacagtgtt	tcttgcccttcctttcctg
